# Causal association between serum bilirubin and ischemic stroke: multivariable Mendelian randomization

**DOI:** 10.4178/epih.e2024070

**Published:** 2024-08-19

**Authors:** Jong Won Shin, Keum Ji Jung, Mikyung Ryu, Jungeun Kim, Heejin Kimm, Sun Ha Jee

**Affiliations:** 1 Department of Epidemiology and Health Promotion, Graduate School of Public Health, Yonsei University, Seoul, Korea; 2 Department of Laboratory Medicine, Asan Medical Center, University of Ulsan College of Medicine, Ulsan, Korea; 3 Institute for Health Promotion, Graduate School of Public Health, Yonsei University, Seoul, Korea; 4 Institute on Aging, Ajou University Medical Center, Suwon, Korea; 5 Basgenbio, Inc., Seoul, Korea

**Keywords:** Bilirubin, Ischemic stroke, Mendelian randomization analysis, Causality

## Abstract

**OBJECTIVES:**

Previous research has predominantly focused on total bilirubin levels without clearly distinguishing between direct and indirect bilirubin. In this study, the differences between these forms were examined, and their potential causal relationships with ischemic stroke were investigated.

**METHODS:**

Two-sample multivariable Mendelian randomization (MVMR) analysis was employed, extracting summary data on bilirubin from the Korean Cancer Prevention Study-II (n=159,844) and the Korean Genome and Epidemiology Study (n=72,299). Data on ischemic stroke were obtained from BioBank Japan (n=201,800). Colocalization analysis was performed, focusing on the *UGT1A1*, *SLCO1B1*, and *SLCO1B3* genes, which are the primary loci associated with serum bilirubin levels.

**RESULTS:**

Crude 2-sample Mendelian randomization analysis revealed a significant negative association between total bilirubin levels and ischemic stroke. However, in MVMR analyses, only indirect bilirubin demonstrated a significant negative association with ischemic stroke (odds ratio, 0.76; 95% confidence interval, 0.59 to 0.98). Colocalization analysis did not identify a shared causal variant between the 3 genetic loci related to indirect bilirubin and the risk of ischemic stroke.

**CONCLUSIONS:**

Our study establishes a causal association between higher genetically determined levels of serum indirect bilirubin and reduced risk of ischemic stroke in an Asian population. Future research should include more in-depth analysis of shared genetic variants between indirect bilirubin and ischemic stroke.

## GRAPHICAL ABSTRACT

[Fig f2-epih-46-e2024070]

## Key Message

This study investigated the causal associations between three forms of serum bilirubin (total, direct, and indirect) and ischemic stroke. Multivariable Mendelian randomization (MVMR) analysis revealed a significant inverse association between indirect bilirubin and the risk of ischemic stroke. These findings contribute to a deeper understanding of the relationship between bilirubin’s antioxidant role and ischemic stroke.

## INTRODUCTION

**Figure 1.**
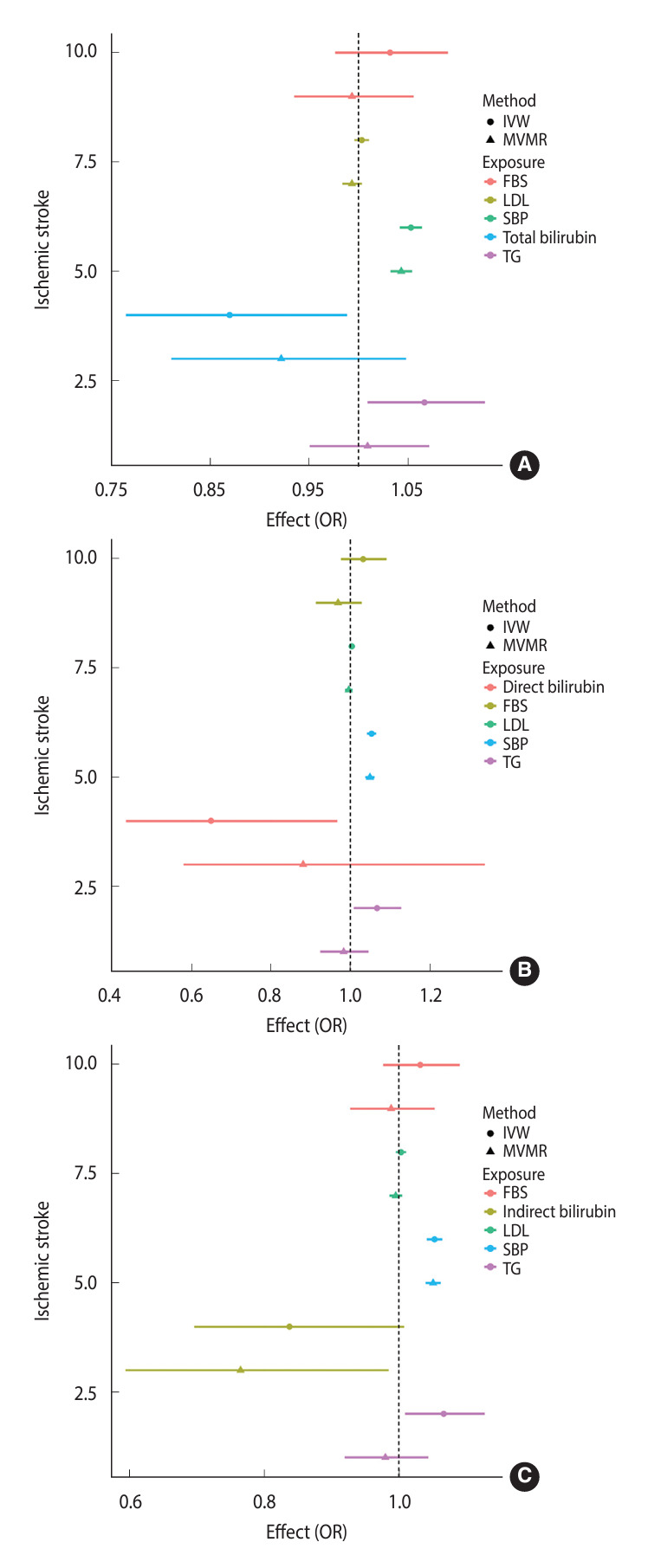
Causal associations of (A) total, (B) direct, and (C) indirect bilirubin levels with ischemic stroke determined using MVMR. OR, odds ratio; IVW, inverse-variance weighted; MVMR, multivariable Mendelian randomization; FBS, fasting blood sugar; LDL, low-density lipoprotein; SBP, systolic blood pressure; TG, triglyceride.

Stroke is recognized as a disease associated with oxidative stress [1-3]. Several studies have reported that serum bilirubin levels are inversely associated with the risk of stroke in both Western [4,5] and Asian populations [6,7], based on measurements of serum total bilirubin. However, these reports do not differentiate between the levels of direct (conjugated) bilirubin and indirect (unconjugated) bilirubin, which together constitute total bilirubin. Indirect bilirubin represents a large proportion of total bilirubin (reference values: total bilirubin, 0.3-1.0 mg/dL; direct bilirubin, 0.0-0.3 mg/dL; indirect bilirubin, 0.2-0.8 mg/dL). The breakdown of aged red blood cells produces heme and globin, with heme subsequently degraded into iron and biliverdin. During the oxidation of heme to biliverdin (Figure 1), indirect bilirubin functions as a potent endogenous antioxidant [8,9].

The first study to investigate the relationship between total bilirubin and stroke incidence using genetic data was a case-cohort study of 806 patients with stroke among a subcohort of 4,793 participants from the Korean Cancer Prevention Study-II (KCPS-II). This study employed one-sample Mendelian randomization (MR) analysis and revealed a negative association between total bilirubin levels and the overall incidence of stroke (hazard ratio [HR], 0.63; 95% confidence interval [CI], 0.30 to 1.36). However, the results were not statistically significant (p=0.240) [10]. A second study used 2-sample MR analysis to examine the relationship between total bilirubin levels and stroke risk, utilizing data from the Korean Genome and Epidemiology Study (KoGES; n=25,406) and the KCPS-II (n=14,541) [10]. The findings indicated a significant causal link between higher total bilirubin levels and a lower risk of stroke in the Korean population, with a stronger association observed for ischemic stroke (odds ratio [OR], 0.302) than total stroke (OR, 0.481) [11]. However, when considering serum total bilirubin alone, the evidence was insufficient to indicate a reduction in stroke risk attributable to the role of bilirubin as an endogenous antioxidant.

In the present study, we examined the types of bilirubin separately to assess their potential impacts as endogenous antioxidants. We also explored the potential significance of causal associations after adjusting for multiple genetic variables, an analysis not feasible in prior MR studies.

## MATERIALS AND METHODS

We analyzed data on total, direct, and indirect bilirubin from the KoGES (n=72,299) [12] and KCPS-II (n=159,844) biobanks [13], as well as data on ischemic stroke from BioBank Japan (BBJ; n=201,800) [14]. These data were utilized in 2-sample multivariable Mendelian randomization (MVMR). This method of analysis was employed to adjust for factors related to stroke, such as blood pressure, fasting blood sugar (FBS), and serum lipid levels.

### Genetic instruments for serum bilirubin (G-X)

Genetic instrumental variables for serum bilirubin were identified using 2 Korean biobanks, KCPS-II and KoGES [12,13]. The selection of instrumental variables for MR analysis adhered to the following criteria. First, cases were required to demonstrate a p-value smaller than the genome-wide significance level identified in the study (p<5×10^-8^). Second, the minor allele frequency (MAF) had to exceed 0.01. Third, single nucleotide polymorphisms (SNPs) with a linkage disequilibrium relationship were excluded (clumping criterion: r^2^ < 0.001). Finally, palindromic SNPs were excluded from the analysis if the MAF was greater than 0.42.

### Bilirubin measurement

Bilirubin levels were measured using an automated chemistry analyzer (AU5800; Beckman Coulter, Seoul, Korea). Total bilirubin levels are determined through the reaction of bilirubin with a stabilized diazonium salt, specifically 3,5-dichlorophenyldiazonium tetrafluoroborate (DPD), resulting in the formation of azobilirubin. Caffeine and surfactants are incorporated to accelerate this reaction. The azobilirubin is then measured based on its absorbance at 570/660 nm, with this absorbance being proportional to the bilirubin concentration in the sample. To correct for any endogenous serum interference, a separate serum blank is also measured. The within-run precision of the assay has a coefficient of variation (CV) of less than 3% or a standard deviation (SD) of ≤ 0.07, while the total precision maintains a CV of less than 5% or an SD of ≤ 0.10.

The measurement of direct bilirubin employs a modified version of the classical method developed by Coolidge [14]. In this method, direct (conjugated) bilirubin reacts with DPD in an acidic environment to produce azobilirubin. The serum concentration of direct bilirubin is proportional to the intensity of the azobilirubin color, which is measured at 540/600 nm. The within-run precision for direct bilirubin measurements displays a CV of less than 7% or an SD of ≤ 0.07, while the total precision exhibits a CV of less than 8% or an SD of ≤ 0.21.

Indirect bilirubin is not measured, but rather is calculated as the difference between total bilirubin and direct bilirubin.

#### Genetic associations of SNPs with ischemic stroke (G-Y)

The summary data used for ischemic stroke were obtained from BBJ [15], a Japanese biobank containing data from 201,800 patients across 66 hospitals nationwide. These patients were enrolled in the BBJ registry between 2003 and 2008.

#### MR

In this study, for 2-sample MR, G-X data on exposure were obtained from KCPS-II and KoGES, while G-Y data on outcome were sourced from BBJ. The coefficient was estimated using the inverse-variance weighted (IVW) method, assuming that all selected SNPs were valid instruments. The coefficient for each SNP was calculated using the Wald ratio method, and these were then combined using the IVW approach.

MVMR analysis was conducted, controlling for genetic variables including triglycerides (TG), low-density lipoprotein (LDL) cholesterol, high-density lipoprotein (HDL) cholesterol, systolic blood pressure (SBP), and FBS, which are recognized as major risk factors for stroke. To confirm the validity of the instrumental variables, the F-value for each variable was reported in the respective models.

#### Colocalization analysis

Colocalization using the coloc method was used to quantify the probability of a shared genetic variant between bilirubin-related variants and ischemic stroke (H4) across 3 genes: UDP glucuronosyltransferase family 1 member A1 (*UGT1A1*) and solute carrier organic anion transporter family members 1B1 (*SLCO1B1*) and 1B3 (*SLCO1B3*). This analysis was based on genetic variants within 500 kb upstream and downstream of *UGT1A1*, *SLCO1B1*, and *SLCO1B3*.

Analyses were conducted using the 2-sample MR package in R version 3.6.0 (R Project for Statistical Computing, Vienna, Austria).

### Ethics statement

The study protocol received approval from the Institutional Review Board of Severance Hospital (approval No. 4-2011-0277).

## RESULTS

This study categorized bilirubin into total, direct, and indirect forms and employed MR analysis to investigate their associations with the risk of ischemic stroke. Summary data for bilirubin levels were extracted from KoGES and KCPS-II, while summary data for ischemic stroke were obtained from BBJ.

Table 1 presents the crude 2-sample MR and MVMR results regarding the effect of total bilirubin on ischemic stroke. Within the KoGES dataset, the crude 2-sample MR analysis revealed a significant negative association between total bilirubin and ischemic stroke (OR, 0.86; 95% CI, 0.75 to 0.98). Additionally, the MVMR analyses that were individually adjusted for LDL, HDL, and TG yielded significant results. For the KCPS-II dataset, the crude 2-sample MR results and the MVMR model adjusted for LDL showed borderline significant results, while the MVMR models controlled for HDL and TG demonstrated significance. In each remaining model, a negative association between total bilirubin and ischemic stroke was observed, but it did not reach statistical significance (Figure 1A). For total bilirubin and the variables used in the models in Table 1, the F-statistics were all above 10, except for SBP. This suggests that the assumption of a valid instrumental variable was met (Supplementary Material 1).

Table 2 presents the crude 2-sample MR and MVMR results regarding the impact of direct bilirubin on ischemic stroke. Analysis of the KoGES data revealed a significant negative association in the crude 2-sample MR only (p=0.049). In contrast, the KCPS-II data analysis demonstrated significant findings in the crude 2-sample MR and the MVMR models individually controlled for LDL, HDL, and TG. The remaining models demonstrated negative relationships, but these findings were not statistically significant (Figure 1B). Except for SBP, the F-values for direct bilirubin and the control variables used in the models in Table 2 were all above 10, fulfilling the criteria for an instrumental variable (Supplementary Material 2).

Table 3 presents the crude 2-sample MR and MVMR results regarding the impact of indirect bilirubin on ischemic stroke. The KoGES data indicated a significant negative association in the crude 2-sample MR analysis only (p=0.025). Conversely, in the KCPS-II data, crude 2-sample MR yielded a borderline significant finding. However, the MVMR analyses that were individually adjusted for LDL, HDL, and TG demonstrated significant associations. Furthermore, the MVMR analysis that concurrently adjusted for all 3 of these variables also exhibited significance (p=0.035; Figure 1C). The F-statistics for indirect bilirubin and the control variables used in the models in Table 3 were all greater than 10, apart from SBP, aligning with the assumption of instrumental variables (Supplementary Material 3).

In the models presented in Tables 1-3, among all controlled variables (that is, those other than bilirubin), only SBP consistently showed a significant causal relationship with the risk of ischemic stroke. Conversely, LDL, TG, and FBS did not display significance.

The colocalization analysis did not reveal a shared causal variant between indirect bilirubin and the risk of ischemic stroke, based on the examination of 3 genes: *UGT1A1*, *SLCO1B1*, and *SLCO1B3* (Supplementary Material 4). Further research should involve an in-depth analysis of genes beyond those studied here. However, summarizing the current findings, a weak causal relationship appeared between indirect bilirubin levels and ischemic stroke. The association observed in prior MR research is likely attributable to pleiotropy (PP.H3=74.9%) (Supplementary Material 5).

The genetic associations of indirect bilirubin, direct bilirubin, and total bilirubin levels with ischemic stroke are detailed in the provided tables. Each table lists the SNP identifiers, effect alleles (A1), other alleles (A2), beta coefficients for bilirubin levels and ischemic stroke (beta.x, beta.y), effect allele frequencies (eaf.x), standard errors (se.x, se.y), and p-values (pval.x, pval.y). These data underpin the MR analysis performed to explore the potential causal relationships between bilirubin subtypes and ischemic stroke (Supplementary Materials 6-8).

The results of the sensitivity analysis for MR between bilirubin levels and stroke risk are presented in Supplementary Material 9. This table details findings obtained using various methods, including MR Egger, weighted median, and weighted mode techniques, for each bilirubin subgroup.

## DISCUSSION

In this study, we demonstrated that genetically determined serum levels of bilirubin (total, direct, and indirect) were causally and inversely associated with the risk of ischemic stroke in an Asian population. Notably, indirect bilirubin alone exhibited a strong protective effect in MVMR analysis, which controlled for genetic factors related to ischemic stroke.

This study utilized 2-sample MR to explore the causal relationships between genetically determined levels of circulating serum bilirubin (total, direct, and indirect) and the risk of ischemic stroke. Furthermore, the analysis accounted for genetic confounders including TG, cholesterol (both HDL and LDL), SBP, and FBS.

Interestingly, when controlling for genetic variables such as TG, HDL-cholesterol and LDL-cholesterol, SBP, and FBS, higher levels of indirect bilirubin were associated with a reduced risk of ischemic stroke. In contrast, a somewhat weaker association was observed for direct bilirubin. These research findings are anticipated to contribute to a better understanding of the mechanisms related to oxidative stress.

In the metabolic breakdown of bilirubin, heme is degraded by heme oxygenase, yielding carbon monoxide, iron, and biliverdin [16,17]. Within normal, healthy liver cells, biliverdin is subsequently converted into bilirubin by biliverdin reductase A (BVRA). Biliverdin reductase exists as 2 isoenzymes: BVRA and biliverdin reductase B, which produce bilirubin Ixα and bilirubin Ixβ, respectively [16]. BVRA plays a key role in the production of bilirubin Ixα in adults, while bilirubin Ixβ is predominantly found during fetal development. Bilirubin Ixα is insoluble and attaches to albumin in the bloodstream, forming indirect or unconjugated bilirubin. It is then transported to the liver, where it undergoes conjugation [16].

Afterward, bilirubin is conjugated in hepatocytes by the *UGT1A1* UDP-glucuronosyltransferase enzyme, producing direct bilirubin (also known as conjugated bilirubin), which is excreted in bile and eventually reaches the intestines [17,18].

Conjugated bilirubin is isolated by intestinal bacteria and reduced to urobilinogen. The enzymes and active sites involved in the metabolism of bilirubin differ at each stage of the process. Since the clinical presentations associated with changes in these levels vary markedly, we anticipated that subclassifying total bilirubin into direct and indirect forms would substantially influence the findings and directions of research.

In 2-sample MR, exposure variables and outcome data are extracted from 2 independent biobanks. This approach is beneficial when exposure and outcome information are not available from the same biobank. In this study, serum bilirubin data were obtained from the KoGES and KCPS-II databases. For ischemic stroke data, MR analysis was conducted using corresponding Asian data from BBJ.

Furthermore, utilizing multiple samples increases the overall sample size, thus improving the precision of causal effect estimates. Consequently, we not only verified the causal relationship between bilirubin levels and reduced stroke risk, as demonstrated previously in Korean cases [10,11], but also confirmed this relationship regarding ischemic stroke risk in an Asian population using Japanese data.

Evidence from numerous observational studies in humans suggests a strong inverse association between serum bilirubin levels and cardiovascular disease. Bilirubin exhibits antioxidant functions, such as scavenging reactive oxygen species (ROS) and inhibiting nicotinamide adenine dinucleotide phosphate (NADPH) oxidase activity, thus reducing oxidative stress. This stress is key to the pathogenesis and progression of atherosclerosis [19,20].

Additionally, serum total bilirubin concentration has been shown to be negatively associated with arteriosclerosis in Chinese men [21]. Similarly, in a German population, serum bilirubin levels were inversely associated with coronary artery calcification and cardiovascular disease [22]. In a Chinese population, lower serum total bilirubin levels were associated with an increased risk of subclinical cerebral infarction, which in turn raised the risk of transient ischemic attack, symptomatic stroke, and cardiovascular disease [23].

Regarding stroke risk, a cross-sectional study of Americans conducted from 1999 to 2004, known as the National Health and Nutrition Examination Survey, reported an inverse association between serum total bilirubin levels and adverse stroke outcomes [24]. In a Korean population, serum bilirubin concentration was found to be negatively correlated with ischemic stroke in men [6].

Additionally, several experimental studies corroborate the findings of this study. When bilirubin acts as an ROS scavenger, indirect bilirubin (albumin-bound unconjugated bilirubin) is oxidized to biliverdin, its non-toxic metabolic precursor. Biliverdin is subsequently recycled back to bilirubin by biliverdin reductase [20,25,26]. *In vitro* studies have demonstrated that indirect bilirubin is oxidized by reactive species such as superoxide anion, hydroxyl radical, and hydroperoxyl. Furthermore, unconjugated bilirubin protects albumin from oxidative damage by these species, as well as by peroxynitrite. Indirect bilirubin can act as an antioxidant by directly scavenging ROS [20,26,27]. Additionally, indirect bilirubin has been found to inhibit the activity of NADPH oxidase—a major source of ROS in the vascular system—both *in vitro* and *in vivo*. These findings suggest that indirect bilirubin reduces ROS production [19,28]. Moreover, bilirubin has been observed to interact with other antioxidants, resulting in synergistic inhibition of lipid peroxidation [29,30]. Collectively, these experimental results suggest that indirect bilirubin can function as an antioxidant by synergistically interacting with other antioxidants to scavenge ROS, inhibit NADPH oxidase activity, and suppress lipid oxidation.

Bilirubin remains a key indicator under study today, as it has been associated with the risk of diseases related to oxidative stress in humans. These include cardiovascular disease, stroke, diabetes, metabolic syndrome, certain cancers, and autoimmune diseases [30,31]. Based on the present findings, further research into other oxidative stress-related diseases could be pursued, utilizing the detailed bilirubin index and specific bilirubin levels while controlling for various genetic variables.

This study had both strengths and limitations. A key strength is the use of bilirubin data from the Korean biobanks KCPS-II and KoGES for 2-sample MR analysis, coupled with the employment of large-scale stroke data from the Japanese biobank BBJ. Furthermore, this study is the first to investigate the effects of direct and indirect bilirubin in addition to total bilirubin, setting it apart from previous research. However, a limitation of this study is its exclusive focus on ischemic stroke, thus omitting total stroke and hemorrhagic stroke from the analysis. However, in prior studies examining the relationship between bilirubin levels and stroke, a significant association was observed only with ischemic stroke. Finally, we recognize the potential for inconsistent results between the KoGES and KCPS-II cohorts. Inconsistencies may arise from differences in exposure data, which could be influenced by variations in baseline characteristics, demographic features, and changes in health status in the cohorts. Given these limitations, the present research findings must be interpreted with caution, especially in light of the issue of multiple comparisons.

In conclusion, this study provides causal evidence that indirect bilirubin is a significant protective factor against the risk of ischemic stroke. However, when comparing the effects of SBP and indirect bilirubin—both identified as causal factors, albeit acting in opposite directions—indirect bilirubin exhibited a much wider CI. This suggests that the reliability and statistical significance of the estimated results are not robust. Future research should therefore aim to further elucidate the protective role of indirect bilirubin in ischemic stroke.

## Figures and Tables

**Figure f2-epih-46-e2024070:**
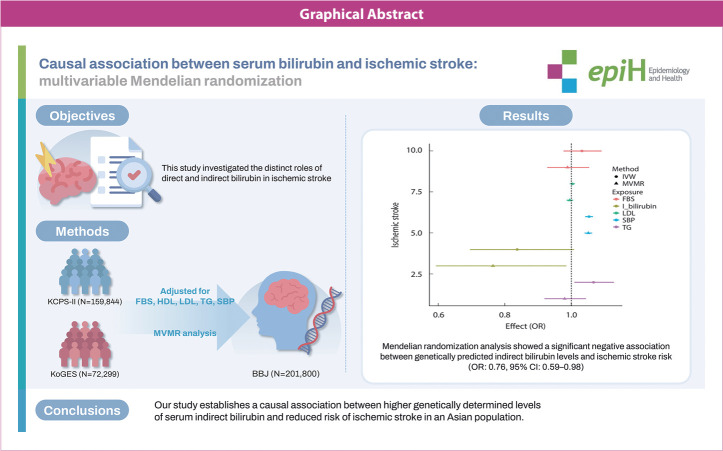


**Table 1.** Causal effect of total bilirubin on ischemic stroke

Variables	Ischemic stroke (BBJ)			
	Total bilirubin (KoGES)	p-value	Total bilirubin (KCPS-II)	p-value
Crude 2-sample MR	0.86 (0.75, 0.98)	0.029	0.87 (0.77, 0.99)	0.065
MVMR				
Adjusted for LDL	0.86 (0.76, 0.96)	0.010	0.89 (0.79, 1.01)	0.067
Adjusted for HDL	0.82 (0.73, 0.93)	0.002	0.88 (0.79, 0.99)	0.033
Adjusted for TG	0.99 (0.99, 1.00)	0.054	0.87 (0.78, 0.98)	0.017
Adjusted for LDL and HDL	0.96 (0.75, 1.23)	0.752	0.95 (0.81, 1.12)	0.526
Adjusted for LDL and TG	0.97 (0.76, 1.23)	0.791	0.91 (0.79, 1.05)	0.201
Adjusted for HDL and TG	0.84 (0.65, 1.08)	0.175	0.94 (0.82, 1.09)	0.422
Adjusted for LDL, HDL, and TG	0.99 (0.78, 1.25)	0.907	0.94 (0.82, 1.08)	0.368
Adjusted for LDL, HDL, TG, and SBP	0.93 (0.76, 1.15)	0.514	0.95 (0.84, 1.08)	0.466
Adjusted for LDL, TG, and SBP	0.87 (0.64, 1.19)	0.389	0.92 (0.81, 1.05)	0.243
Adjusted for LDL, TG, SBP, and FBS	0.90 (0.68, 1.20)	0.488	0.92 (0.81, 1.05)	0.214
Adjusted for LDL, TG, and FBS	0.83 (0.65, 1.05)	0.125	0.91 (0.79, 1.04)	0.172

Values are presented as odds ratio (95% confidence interval) by IVW method. BBJ, Biobank Japan; KoGES, Korean Genome Epidemiologic Study; KCPS-II, Korean Cancer Prevention Study-II; MR, Mendelian randomization; MVMR, multivariable Mendelian randomization; LDL, low-density lipoprotein; HDL, high-density lipoprotein; TG, triglyceride; SBP, systolic blood pressure; FBS, fasting blood sugar; IVW, inverse-variance weighted.

**Table 2.** Causal effect of direct bilirubin on ischemic stroke

Variables	Ischemic stroke (BBJ)			
	Direct bilirubin (KoGES)	p-value	Direct bilirubin (KCPS-II)	p-value
Crude 2-sample MR	0.67 (0.45, 1.00)	0.049	0.65 (0.43, 0.97)	0.033
MVMR				
Adjusted for LDL	0.94 (0.55, 1.60)	0.817	0.64 (0.46, 0.89)	0.009
Adjusted for HDL	0.90 (0.52, 1.56)	0.720	0.65 (0.47, 0.91)	0.012
Adjusted for TG	0.80 (0.47, 1.36)	0.414	0.64 (0.46, 0.88)	0.006
Adjusted for LDL and HDL	0.81 (0.37, 1.77)	0.599	0.72 (0.43, 1.19)	0.203
Adjusted for LDL and TG	1.02 (0.46, 2.25)	0.956	0.91 (0.56, 1.46)	0.694
Adjusted for HDL and TG	1.34 (0.73, 2.46)	0.347	0.84 (0.47, 1.52)	0.573
Adjusted for LDL, HDL, and TG	0.72 (0.41, 1.27)	0.262	0.92 (0.59, 1.45)	0.730
Adjusted for LDL, HDL, TG, and SBP	0.88 (0.49, 1.60)	0.685	0.94 (0.61, 1.44)	0.708
Adjusted for LDL, TG, and SBP	0.97 (0.53, 1.80)	0.935	0.88 (0.57, 1.36)	0.556
Adjusted for LDL, TG, SBP, and FBS	1.63 (0.96, 2.77)	0.073	0.88 (0.58, 1.34)	0.552
Adjusted for LDL, TG, and FBS	1.41 (0.85, 2.31)	0.178	0.91 (0.58, 1.44)	0.699

Values are presented as odds ratio (95% confidence interval) by IVW method. BBJ, Biobank Japan; KoGES, Korean Genome Epidemiologic Study; KCPS-II, Korean Cancer Prevention Study-II; MR, Mendelian randomization; MVMR, multivariable Mendelian randomization; LDL, low-density lipoprotein; HDL, high-density lipoprotein; TG, triglyceride; SBP, systolic blood pressure; FBS, fasting blood sugar; IVW, inverse-variance weighted.

**Table 3.** Causal effect of indirect bilirubin on ischemic stroke

Variables	Ischemic stroke (BBJ)			
	Indirect bilirubin (KoGES)	p-value	Indirect bilirubin (KCPS-II)	p-value
Crude 2-sample MR	0.81 (0.68, 0.97)	0.025	0.84 (0.70, 1.01)	0.060
MVMR				
Adjusted for LDL	0.81 (0.65, 1.02)	0.080	0.84 (0.72, 0.98)	0.029
Adjusted for HDL	0.88 (0.71, 1.08)	0.226	0.85 (0.72, 1.00)	0.049
Adjusted for TG	0.85 (0.69, 1.06)	0.153	0.82 (0.70, 0.96)	0.015
Adjusted for LDL and HDL	0.94 (0.62, 1.44)	0.794	0.87 (0.68, 1.11)	0.260
Adjusted for LDL and TG	0.98 (0.69, 1.39)	0.899	0.74 (0.55, 0.99)	0.043
Adjusted for HDL and TG	0.99 (0.63, 1.57)	0.987	0.87 (0.65, 1.16)	0.345
Adjusted for LDL, HDL, and TG	0.79 (0.58, 1.06)	0.119	0.74 (0.56, 0.98)	0.035
Adjusted for LDL, HDL, TG, and SBP	0.95 (0.70, 1.30)	0.765	0.78 (0.60, 1.01)	0.066
Adjusted for LDL, TG, and SBP	1.14 (0.73, 1.78)	0.572	0.77 (0.59, 0.99)	0.050
Adjusted for LDL, TG, SBP, and FBS	0.99 (0.99, 1.00)	0.430	0.76 (0.59, 0.98)	0.039
Adjusted for LDL, TG, and FBS	0.93 (0.60, 1.42)	0.738	0.75 (0.57, 0.98)	0.035

Values are presented as odds ratio (95% confidence interval) by IVW method. BBJ, Biobank Japan; KoGES, Korean Genome Epidemiologic Study; KCPS-II, Korean Cancer Prevention Study-II; MR, Mendelian randomization; MVMR, multivariable Mendelian randomization; LDL, low-density lipoprotein; HDL, high-density lipoprotein; TG, triglyceride; SBP, systolic blood pressure; FBS, fasting blood sugar; IVW, inverse-variance weighted.

## Supplementary materials

Supplementary Material 1. Causal effect of total bilirubin on ischemic stroke

Supplementary Material 2. Causal effect of direct bilirubin on ischemic stroke

Supplementary Material 3. Causal effect of indirect bilirubin on ischemic stroke

Supplementary Material 4. Co-localization analysis on total and indirect bilirubin and ischemic stroke

Supplementary Material 5. List of 79 SNPs and association with indirect bilirubin and ischemic stroke

Supplementary Material 6. List of 65 SNPs and association with direct bilirubin and ischemic stroke

Supplementary Material 7. List of 99 SNPs and association with total bilirubin and ischemic stroke

Supplementary Material 8. Sensitivity analysis in MR regarding bilirubin and ischemic stroke

Supplementary Material 9. LocusZoom plots illustrating no evidence of genetic colocalization between indirect bilirubin at the *SLCO1B3* gene locus and ischemic stroke risk at the *SLCO1B3* gene locus.
