# Supplementary material for: Causal association between serum bilirubin and ischemic stroke: multivariable Mendelian randomization
Source: Epidemiol Health. 2024 Aug 19;46:e2024070. doi: 10.4178/epih.e2024070 (PMC11826012; doi:10.4178/epih.e2024070)
Supplement: Supplementary Material 1. — Causal effect of total bilirubin on ischemic stroke [file epih-46-e2024070-Supplementary-1.docx]

Supplementary Material 1. Causal effect of total bilirubin on ischemic stroke

|  | Total bilirubin(KoGES), Ischemic stroke(BBJ) | | | | | | Total bilirubin(KCPS-II), Ischemic stroke(BBJ) | | | | | |
| --- | --- | --- | --- | --- | --- | --- | --- | --- | --- | --- | --- | --- |
|  | F value | | | | | | F value | | | | | |
|  | t.bil | LDL | HDL | TG | SBP | FBS | t.bil | LDL | HDL | TG | SBP | FBS |
| Crude two sample MR | 418.2 |  |  |  |  |  | 375.6 |  |  |  |  |  |
| MVMR |  |  |  |  |  |  |  |  |  |  |  |  |
| Adjusted for LDL | 150.2 | 31.7 |  |  |  |  | 150.2 | 31.7 |  |  |  |  |
| Adjusted for HDL | 102.5 |  | 73.3 |  |  |  | 207.0 |  | 47.6 |  |  |  |
| Adjusted for TG* | 141.4 |  |  | 84.7 |  |  | 128.8 |  |  | 90.9 |  |  |
| Adjusted for LDL and HDL | 42.0 | 44.1 | 50.7 |  |  |  | 105.9 | 21.3 | 32.8 |  |  |  |
| Adjusted for LDL and TG | 53.3 | 56.0 |  | 58.3 |  |  | 116.4 | 23.6 |  | 69.4 |  |  |
| Adjusted for HDL and TG | 34.2 |  | 21.5 | 23.3 |  |  | 154.8 |  | 12.5 | 17.9 |  |  |
| Adjusted for LDL, HDL and TG | 25.3 | 17.9 | 17.3 | 19.1 |  |  | 121.8 | 13.7 | 10.4 | 14.9 |  |  |
| Adjusted for LDL, HDL, TG and SBP | 34.5 | 15.6 | 14.5 | 15.3 | 4.6 |  | 102.4 | 11.3 | 8.0 | 10.4 | 8.2 |  |
| Adjusted for LDL, TG and SBP | 30.5 | 22.9 |  | 49.3 | 6.2 |  | 95.9 | 17.7 |  | 43.7 | 9.0 |  |
| Adjusted for LDL, TG, SBP and FSG | 27.2 | 19.3 |  | 41.9 | 5.1 | 9.6 | 80.2 | 14.7 |  | 36.8 | 7.5 | 11.3 |
| Adjusted for LDL, TG, and FSG | 30.7 | 22.9 |  | 52.2 |  | 10.8 | 93.0 | 19.0 |  | 50.3 |  | 13.9 |

KoGES, Korean Genome Epidemiologic Study; KCPS-II, Korean Cancer Prevention Study-II; BBJ, Biobank of Japan; MVMR, multivariable mendelian randomization; t.bil, total bilirubin; LDL, low density lipoprotein; HDL, high density lipoprotein; TG, triglyceride; SBP, systolic blood pressure; FBS, fasting serum glucose
