# Supplementary material for: Causal association between serum bilirubin and ischemic stroke: multivariable Mendelian randomization
Source: Epidemiol Health. 2024 Aug 19;46:e2024070. doi: 10.4178/epih.e2024070 (PMC11826012; doi:10.4178/epih.e2024070)
Supplement: Supplementary Material 2. — Causal effect of direct bilirubin on ischemic stroke [file epih-46-e2024070-Supplementary-2.docx]

Supplementary Material 2. Causal effect of direct bilirubin on ischemic stroke

|  | Direct bilirubin(KoGES), Ischemic stroke(BBJ) | | | | | | Direct bilirubin(KCPS-II), Ischemic stroke(BBJ) | | | | | |
| --- | --- | --- | --- | --- | --- | --- | --- | --- | --- | --- | --- | --- |
|  | F value | | | | | | F value | | | | | |
|  | d.bil | LDL | HDL | TG | SBP | FBS | d.bil | LDL | HDL | TG | SBP | FBS |
| Crude two sample MR | 299.6 |  |  |  |  |  | 351.7 |  |  |  |  |  |
| MVMR |  |  |  |  |  |  |  |  |  |  |  |  |
| Adjusted for LDL | 87.5 | 55.2 |  |  |  |  | 138.7 | 36.9 |  |  |  |  |
| Adjusted for HDL | 59.0 |  | 83.1 |  |  |  | 163.2 |  | 56.5 |  |  |  |
| Adjusted for TG* | 59.8 |  |  | 79.8 |  |  | 86.9 |  |  | 96.6 |  |  |
| Adjusted for LDL and HDL | 41.3 | 40.4 | 34.1 |  |  |  | 62.6 | 23.1 | 37.2 |  |  |  |
| Adjusted for LDL and TG | 37.5 | 48.2 |  | 61.9 |  |  | 58.0 | 27.0 |  | 75.0 |  |  |
| Adjusted for HDL and TG | 50.3 |  | 46.5 | 25.5 |  |  | 52.6 |  | 17.5 | 19.9 |  |  |
| Adjusted for LDL, HDL and TG | 33.4 | 33.3 | 19.2 | 28.8 |  |  | 59.6 | 14.4 | 13.3 | 19.5 |  |  |
| Adjusted for LDL, HDL, TG and SBP | 30.7 | 24.5 | 28.4 | 17.2 | 4.4 |  | 47.5 | 11.9 | 9.8 | 12.9 | 9.1 |  |
| Adjusted for LDL, TG and SBP | 46.8 | 41.8 |  | 24.1 | 6.4 |  | 45.4 | 19.8 |  | 46.0 | 10.1 |  |
| Adjusted for LDL, TG, SBP and FSG | 35.6 | 31.7 |  | 18.3 | 4.7 | 11.3 | 37.1 | 16.1 |  | 38.4 | 8.2 | 12.9 |
| Adjusted for LDL, TG, and FSG | 39.2 | 35.7 |  | 22.1 |  | 15.2 | 45.4 | 21.1 |  | 54.8 |  | 16.1 |

KoGES, Korean Genome Epidemiologic Study; KCPS-II, Korean Cancer Prevention Study-II; BBJ, Biobank of Japan; MVMR, multivariable mendelian randomization; d.bil, direct; LDL, low density lipoprotein; HDL, high density lipoprotein; TG, triglyceride; SBP, systolic blood pressure; FBS, fasting serum glucose
