# Supplementary material for: Causal association between serum bilirubin and ischemic stroke: multivariable Mendelian randomization
Source: Epidemiol Health. 2024 Aug 19;46:e2024070. doi: 10.4178/epih.e2024070 (PMC11826012; doi:10.4178/epih.e2024070)
Supplement: Supplementary Material 3. — Causal effect of indirect bilirubin on ischemic stroke [file epih-46-e2024070-Supplementary-3.docx]

Supplementary Material 3. Causal effect of indirect bilirubin on ischemic stroke

|  | Indirect bilirubin(KoGES), Ischemic stroke(BBJ) | | | | | | Indirect bilirubin(KCPS-II), Ischemic stroke(BBJ) | | | | | |
| --- | --- | --- | --- | --- | --- | --- | --- | --- | --- | --- | --- | --- |
|  | F value | | | | | | F value | | | | | |
|  | i.bil | LDL | HDL | TG | SBP | FBS | i.bil | LDL | HDL | TG | SBP | FBS |
| Crude two sample MR | 373.4 |  |  |  |  |  | 379.7 |  |  |  |  |  |
| MVMR |  |  |  |  |  |  |  |  |  |  |  |  |
| Adjusted for LDL | 104.9 | 34.6 |  |  |  |  | 173.6 | 33.6 |  |  |  |  |
| Adjusted for HDL | 79.8 |  | 82.2 |  |  |  | 187.0 |  | 52.5 |  |  |  |
| Adjusted for TG* | 80.6 |  |  | 88.2 |  |  | 110.0 |  |  | 94.3 |  |  |
| Adjusted for LDL and HDL | 23.9 | 22.3 | 61.8 |  |  |  | 81.5 | 22.6 | 34.7 |  |  |  |
| Adjusted for LDL and TG | 47.5 | 64.6 |  | 61.7 |  |  | 49.7 | 27.4 |  | 67.9 |  |  |
| Adjusted for HDL and TG | 28.1 |  | 36.3 | 34.8 |  |  | 68.7 |  | 16.5 | 18.8 |  |  |
| Adjusted for LDL, HDL and TG | 36.4 | 17.5 | 26.7 | 28.7 |  |  | 52.9 | 10.8 | 15.7 | 15.4 |  |  |
| Adjusted for LDL, HDL, TG and SBP | 33.2 | 15.5 | 17.3 | 18.1 | 5.6 |  | 43.7 | 12.8 | 8.3 | 11.1 | 9.5 |  |
| Adjusted for LDL, TG and SBP | 27.9 | 22.6 |  | 51.5 | 8.1 |  | 40.8 | 20.8 |  | 44.1 | 10.2 |  |
| Adjusted for LDL, TG, SBP and FSG | 38.1 | 17.8 |  | 41.3 | 6.4 | 11.7 | 33.7 | 17.1 |  | 36.1 | 8.3 | 11.2 |
| Adjusted for LDL, TG, and FSG | 25.1 | 21.7 |  | 48.9 |  | 15.2 | 38.9 | 21.4 |  | 48.1 |  | 14.4 |

KoGES, Korean Genome Epidemiologic Study; KCPS-II, Korean Cancer Prevention Study-II; BBJ, Biobank of Japan; MVMR, multivariable mendelian randomization; i.bil, indirect bilirubin; LDL, low density lipoprotein; HDL, high density lipoprotein; TG, triglyceride; SBP, systolic blood pressure; FBS, fasting serum glucose
