# Supplementary material for: Causal association between serum bilirubin and ischemic stroke: multivariable Mendelian randomization
Source: Epidemiol Health. 2024 Aug 19;46:e2024070. doi: 10.4178/epih.e2024070 (PMC11826012; doi:10.4178/epih.e2024070)
Supplement: Supplementary Material 4. — Co-localization analysis on total and indirect bilirubin and ischemic stroke [file epih-46-e2024070-Supplementary-4.docx]

Supplementary Material 4. Co-localization analysis on total and indirect bilirubin and ischemic stroke

| Total bilirubin | PP.H0 | PP.H1 | PP.H2 | PP.H3 | PP.H4 |
| --- | --- | --- | --- | --- | --- |
| *UGT1A1* | 0.000 | 0.976 | 0.000 | 0.051 | 0.003 |
| *SLCO1B1* | 0.991 | 0.004 | 0.000 | 0.000 | 0.000 |
| *SLCO1B3* | 0.000 | 0.250 | 0.000 | 0.749 | 0.00179 |
| Indirect bilirubin |  |  |  |  |  |
| *UGT1A1* | 0.000 | 0.964 | 0.000 | 0.033 | 0.003 |
| *SLCO1B1* | 0.986 | 0.007 | 0.006 | 0.000 | 0.000 |
| *SLCO1B3* | 0.000 | .249 | 0.000 | 0.749 | 0.00178 |

PP: Posterior probability
