# Supplementary material for: Causal association between serum bilirubin and ischemic stroke: multivariable Mendelian randomization
Source: Epidemiol Health. 2024 Aug 19;46:e2024070. doi: 10.4178/epih.e2024070 (PMC11826012; doi:10.4178/epih.e2024070)
Supplement: Supplementary Material 5. — List of 79 SNPs and association with indirect bilirubin and ischemic stroke [file epih-46-e2024070-Supplementary-5.docx]

Supplementary Material 5. List of 79 SNPs and association with indirect bilirubin and ischemic stroke

|  | SNP | A1 | A2 | beta.x | beta.y | eaf.x | se.y | pval.y | se.x | pval.x | exposure |
| --- | --- | --- | --- | --- | --- | --- | --- | --- | --- | --- | --- |
| 1 | rs10169532 | T | C | 0.0265 | 0.0076 | 0.458 | 0.011 | 0.4739 | 0.001 | 1.75E-148 | Indirect bil. |
| 2 | rs10175949 | G | A | 0.0127 | 0.0075 | 0.103 | 0.017 | 0.6637 | 0.002 | 2.51E-14 | Indirect bil. |
| 3 | rs10445033 | G | A | 0.008 | -0.0064 | 0.369 | 0.011 | 0.5687 | 0.001 | 4.38E-14 | Indirect bil. |
| 4 | rs10466790 | T | C | -0.0136 | 0.0241 | 0.051 | 0.022 | 0.2648 | 0.002 | 3.58E-09 | Indirect bil. |
| 5 | rs10743399 | G | A | 0.0407 | -0.0037 | 0.213 | 0.013 | 0.7708 | 0.001 | 1.00E-200 | Indirect bil. |
| 6 | rs10761760 | C | A | -0.0121 | 0.0041 | 0.384 | 0.011 | 0.7015 | 0.001 | 7.21E-31 | Indirect bil. |
| 7 | rs10841651 | T | C | 0.0195 | -0.0317 | 0.162 | 0.015 | 0.0358 | 0.001 | 2.52E-45 | Indirect bil. |
| 8 | rs10899116 | T | C | 0.0069 | 0.0172 | 0.222 | 0.012 | 0.1452 | 0.001 | 2.08E-08 | Indirect bil. |
| 9 | rs11045913 | A | G | -0.0105 | -0.0118 | 0.403 | 0.011 | 0.272 | 0.001 | 7.00E-24 | Indirect bil. |
| 10 | rs114114722 | A | G | 0.0135 | -0.0172 | 0.074 | 0.022 | 0.4266 | 0.002 | 3.95E-12 | Indirect bil. |
| 11 | rs11563068 | A | G | -0.012 | -0.0027 | 0.212 | 0.013 | 0.8276 | 0.001 | 4.47E-22 | Indirect bil. |
| 12 | rs11563102 | A | C | 0.0273 | -0.0116 | 0.06 | 0.023 | 0.6168 | 0.002 | 1.57E-37 | Indirect bil. |
| 13 | rs11563223 | C | T | -0.0148 | 0.0158 | 0.059 | 0.021 | 0.4622 | 0.002 | 7.49E-12 | Indirect bil. |
| 14 | rs11568853 | C | T | 0.0066 | -0.0124 | 0.253 | 0.013 | 0.3454 | 0.001 | 2.09E-08 | Indirect bil. |
| 15 | rs11688580 | A | G | -0.0066 | 0.0124 | 0.36 | 0.012 | 0.2932 | 0.001 | 4.33E-10 | Indirect bil. |
| 16 | rs117011528 | A | G | -0.0285 | 0.0067 | 0.09 | 0.017 | 0.6948 | 0.002 | 1.42E-57 | Indirect bil. |
| 17 | rs117846878 | G | T | -0.0131 | 0.0403 | 0.06 | 0.026 | 0.1259 | 0.002 | 8.86E-10 | Indirect bil. |
| 18 | rs11836987 | C | T | -0.007 | -0.005 | 0.362 | 0.011 | 0.66 | 0.001 | 3.37E-11 | Indirect bil. |
| 19 | rs11890704 | C | T | 0.0085 | 0.0119 | 0.197 | 0.013 | 0.368 | 0.001 | 4.04E-11 | Indirect bil. |
| 20 | rs12052961 | A | G | -0.006 | -0.0098 | 0.447 | 0.011 | 0.3642 | 0.001 | 5.23E-09 | Indirect bil. |
| 21 | rs12228427 | G | A | 0.028 | -0.0066 | 0.086 | 0.019 | 0.7275 | 0.002 | 2.43E-54 | Indirect bil. |
| 22 | rs12308309 | G | C | -0.0085 | 0.0143 | 0.189 | 0.014 | 0.2999 | 0.001 | 6.26E-11 | Indirect bil. |
| 23 | rs12996139 | A | C | 0.0203 | -0.0148 | 0.102 | 0.015 | 0.3199 | 0.002 | 1.30E-33 | Indirect bil. |
| 24 | rs13018934 | G | A | 0.0067 | -0.0109 | 0.405 | 0.011 | 0.3238 | 0.001 | 6.51E-11 | Indirect bil. |
| 25 | rs13030735 | A | C | -0.0266 | 0.0127 | 0.318 | 0.012 | 0.2737 | 0.001 | 9.95E-131 | Indirect bil. |
| 26 | rs13289294 | T | C | 0.0073 | 0.0009 | 0.442 | 0.011 | 0.9343 | 0.001 | 1.33E-12 | Indirect bil. |
| 27 | rs144708372 | T | C | 0.018 | -0.0228 | 0.055 | 0.022 | 0.2955 | 0.002 | 1.22E-15 | Indirect bil. |
| 28 | rs147132866 | C | T | 0.0777 | -0.0469 | 0.052 | 0.026 | 0.0756 | 0.002 | 1.00E-200 | Indirect bil. |
| 29 | rs150741507 | C | T | -0.062 | 0.0278 | 0.067 | 0.024 | 0.2521 | 0.002 | 1.00E-200 | Indirect bil. |
| 30 | rs1597944 | C | T | 0.0728 | 0.0036 | 0.465 | 0.011 | 0.7488 | 0.001 | 1.00E-200 | Indirect bil. |
| 31 | rs1654774 | A | G | 0.0069 | -0.0122 | 0.382 | 0.011 | 0.2686 | 0.001 | 5.74E-11 | Indirect bil. |
| 32 | rs1661052 | G | A | 0.0239 | 0.0585 | 0.09 | 0.021 | 0.0057 | 0.002 | 9.90E-41 | Indirect bil. |
| 33 | rs17866592 | C | T | -0.0142 | -0.0088 | 0.073 | 0.018 | 0.6343 | 0.002 | 4.73E-13 | Indirect bil. |
| 34 | rs17868401 | A | G | 0.0097 | -0.003 | 0.304 | 0.012 | 0.8 | 0.001 | 1.19E-18 | Indirect bil. |
| 35 | rs17869073 | A | C | -0.0069 | 0.0248 | 0.322 | 0.011 | 0.0289 | 0.001 | 3.04E-10 | Indirect bil. |
| 36 | rs180363 | C | T | -0.0078 | -0.012 | 0.181 | 0.016 | 0.4569 | 0.001 | 4.10E-09 | Indirect bil. |
| 37 | rs2068888 | G | A | -0.0079 | 0.0346 | 0.271 | 0.011 | 0.0016 | 0.001 | 4.61E-12 | Indirect bil. |
| 38 | rs2174011 | G | A | -0.0179 | 0.0236 | 0.317 | 0.012 | 0.0484 | 0.001 | 8.82E-61 | Indirect bil. |
| 39 | rs2199766 | G | A | 0.0157 | -0.0058 | 0.434 | 0.011 | 0.5887 | 0.001 | 6.66E-53 | Indirect bil. |
| 40 | rs2242097 | C | T | 0.0464 | -0.009 | 0.262 | 0.013 | 0.5034 | 0.001 | 1.00E-200 | Indirect bil. |
| 41 | rs2293439 | C | A | 0.0069 | 0.0155 | 0.437 | 0.011 | 0.1577 | 0.001 | 2.09E-11 | Indirect bil. |
| 42 | rs2302154 | C | T | 0.0137 | -0.0114 | 0.07 | 0.022 | 0.5973 | 0.002 | 8.42E-12 | Indirect bil. |
| 43 | rs2304776 | T | C | -0.0211 | -0.0016 | 0.338 | 0.012 | 0.8974 | 0.001 | 7.11E-86 | Indirect bil. |
| 44 | rs2844824 | T | A | -0.0057 | -0.0166 | 0.433 | 0.012 | 0.1527 | 0.001 | 2.99E-08 | Indirect bil. |
| 45 | rs28514310 | T | C | 0.0111 | -0.0137 | 0.276 | 0.013 | 0.2835 | 0.001 | 1.84E-22 | Indirect bil. |
| 46 | rs28969670 | C | T | 0.0388 | -0.0096 | 0.07 | 0.024 | 0.6876 | 0.002 | 1.47E-84 | Indirect bil. |
| 47 | rs3764043 | T | C | -0.0077 | 0.0282 | 0.2 | 0.017 | 0.0885 | 0.001 | 1.14E-09 | Indirect bil. |
| 48 | rs379149 | T | C | -0.0096 | 0.0123 | 0.114 | 0.02 | 0.5409 | 0.002 | 2.38E-09 | Indirect bil. |
| 49 | rs4410790 | C | T | -0.0059 | 0.0093 | 0.401 | 0.011 | 0.3962 | 0.001 | 1.41E-08 | Indirect bil. |
| 50 | rs4737010 | G | A | 0.0057 | 0.0121 | 0.463 | 0.011 | 0.2885 | 0.001 | 2.05E-08 | Indirect bil. |
| 51 | rs4972193 | T | C | 0.0058 | 0.0028 | 0.395 | 0.011 | 0.7948 | 0.001 | 3.20E-08 | Indirect bil. |
| 52 | rs55686299 | G | T | 0.0326 | -0.0042 | 0.066 | 0.024 | 0.8614 | 0.002 | 1.42E-57 | Indirect bil. |
| 53 | rs567988934 | G | T | -0.0239 | -0.0062 | 0.056 | 0.026 | 0.8098 | 0.002 | 4.65E-27 | Indirect bil. |
| 54 | rs662799 | G | A | 0.0116 | -0.0181 | 0.296 | 0.011 | 0.1084 | 0.001 | 3.00E-25 | Indirect bil. |
| 55 | rs663705 | T | C | 0.014 | 0.0087 | 0.128 | 0.014 | 0.5294 | 0.002 | 4.98E-20 | Indirect bil. |
| 56 | rs6715829 | T | A | 0.1146 | -0.0219 | 0.097 | 0.017 | 0.2093 | 0.002 | 1.00E-200 | Indirect bil. |
| 57 | rs6717651 | G | A | -0.0072 | 0.0208 | 0.28 | 0.012 | 0.0834 | 0.001 | 1.85E-10 | Indirect bil. |
| 58 | rs6751673 | A | G | 0.0406 | -0.0204 | 0.198 | 0.015 | 0.1813 | 0.001 | 1.00E-200 | Indirect bil. |
| 59 | rs6758317 | T | C | 0.0417 | -0.0069 | 0.128 | 0.016 | 0.6543 | 0.002 | 4.66E-165 | Indirect bil. |
| 60 | rs7260044 | T | C | 0.0061 | -0.0301 | 0.378 | 0.011 | 0.0082 | 0.001 | 7.61E-09 | Indirect bil. |
| 61 | rs7285681 | C | G | 0.0056 | -0.012 | 0.482 | 0.011 | 0.2691 | 0.001 | 3.50E-08 | Indirect bil. |
| 62 | rs72978109 | C | T | 0.0062 | -0.0031 | 0.347 | 0.012 | 0.804 | 0.001 | 7.05E-09 | Indirect bil. |
| 63 | rs7305718 | C | T | -0.0072 | -0.0158 | 0.215 | 0.013 | 0.2124 | 0.001 | 6.39E-09 | Indirect bil. |
| 64 | rs7310077 | G | A | 0.012 | -0.0272 | 0.181 | 0.014 | 0.056 | 0.001 | 9.22E-20 | Indirect bil. |
| 65 | rs73683955 | T | C | -0.0082 | 0.0018 | 0.191 | 0.014 | 0.9013 | 0.001 | 2.00E-10 | Indirect bil. |
| 66 | rs74368849 | A | G | 0.0118 | 0.0008 | 0.078 | 0.02 | 0.9702 | 0.002 | 4.83E-10 | Indirect bil. |
| 67 | rs75544767 | C | T | 0.0131 | 0.0132 | 0.062 | 0.026 | 0.6141 | 0.002 | 4.51E-10 | Indirect bil. |
| 68 | rs7571915 | A | G | 0.0938 | -0.0034 | 0.231 | 0.012 | 0.7852 | 0.001 | 1.00E-200 | Indirect bil. |
| 69 | rs7591535 | T | A | 0.03 | -0.0474 | 0.068 | 0.028 | 0.0919 | 0.002 | 1.27E-49 | Indirect bil. |
| 70 | rs76159953 | C | T | -0.0112 | 0.0634 | 0.075 | 0.028 | 0.023 | 0.002 | 7.62E-09 | Indirect bil. |
| 71 | rs7741443 | T | A | -0.0092 | 0.0405 | 0.119 | 0.018 | 0.0218 | 0.002 | 4.68E-09 | Indirect bil. |
| 72 | rs77768175 | G | A | -0.0104 | -0.1226 | 0.162 | 0.014 | 0 | 0.002 | 7.71E-10 | Indirect bil. |
| 73 | rs79522608 | A | G | -0.0145 | -0.0071 | 0.083 | 0.02 | 0.7263 | 0.002 | 2.94E-15 | Indirect bil. |
| 74 | rs9247 | T | C | 0.0066 | 0.0069 | 0.344 | 0.012 | 0.5484 | 0.001 | 5.88E-10 | Indirect bil. |
| 75 | rs9393734 | T | C | -0.0098 | 0.0336 | 0.145 | 0.016 | 0.0311 | 0.001 | 1.36E-11 | Indirect bil. |
| 76 | rs9646718 | T | C | -0.0212 | 0.0224 | 0.056 | 0.024 | 0.3506 | 0.002 | 7.50E-22 | Indirect bil. |
| 77 | rs9646719 | C | T | -0.0157 | 0.0204 | 0.458 | 0.011 | 0.0579 | 0.001 | 5.54E-53 | Indirect bil. |
| 78 | rs975419 | G | A | 0.008 | 0.015 | 0.145 | 0.017 | 0.3837 | 0.001 | 3.62E-08 | Indirect bil. |
| 79 | rs9873528 | A | G | -0.0058 | 0.0017 | 0.373 | 0.011 | 0.8735 | 0.001 | 2.99E-08 | Indirect bil. |
