# Supplementary material for: Causal association between serum bilirubin and ischemic stroke: multivariable Mendelian randomization
Source: Epidemiol Health. 2024 Aug 19;46:e2024070. doi: 10.4178/epih.e2024070 (PMC11826012; doi:10.4178/epih.e2024070)
Supplement: Supplementary Material 6. — List of 65 SNPs and association with direct bilirubin and ischemic stroke [file epih-46-e2024070-Supplementary-6.docx]

Supplementary Material 6. List of 65 SNPs and association with direct bilirubin and ischemic stroke

|  | SNP | A1 | A2 | beta.x | beta.y | eaf.x | se.y | pval.y | se.x | pval.x | exposure |
| --- | --- | --- | --- | --- | --- | --- | --- | --- | --- | --- | --- |
| 1 | rs10169532 | T | C | 0.0117 | 0.0076 | 0.458 | 0.011 | 0.4739 | 0.001 | 4.68E-95 | Direct bil. |
| 2 | rs10175949 | G | A | 0.0062 | 0.0075 | 0.103 | 0.017 | 0.6637 | 0.001 | 2.57E-11 | Direct bil. |
| 3 | rs10466790 | T | C | -0.0089 | 0.0241 | 0.051 | 0.022 | 0.2648 | 0.001 | 3.48E-12 | Direct bil. |
| 4 | rs10743399 | G | A | 0.0238 | -0.0037 | 0.213 | 0.013 | 0.7708 | 0.001 | 1.00E-200 | Direct bil. |
| 5 | rs10743414 | C | T | -0.0036 | -0.0030 | 0.349 | 0.011 | 0.7937 | 0.001 | 1.21E-09 | Direct bil. |
| 6 | rs10761760 | C | A | -0.0051 | 0.0041 | 0.384 | 0.011 | 0.7015 | 0.001 | 3.70E-18 | Direct bil. |
| 7 | rs10841651 | T | C | 0.0110 | -0.0317 | 0.162 | 0.015 | 0.0358 | 0.001 | 2.71E-46 | Direct bil. |
| 8 | rs11045913 | A | G | -0.0066 | -0.0118 | 0.403 | 0.011 | 0.2720 | 0.001 | 1.75E-30 | Direct bil. |
| 9 | rs114114722 | A | G | 0.0068 | -0.0172 | 0.074 | 0.022 | 0.4266 | 0.001 | 4.50E-10 | Direct bil. |
| 10 | rs11563102 | A | C | 0.0132 | -0.0116 | 0.060 | 0.023 | 0.6168 | 0.001 | 9.36E-29 | Direct bil. |
| 11 | rs11563214 | G | T | -0.0059 | 0.0024 | 0.233 | 0.012 | 0.8409 | 0.001 | 1.14E-18 | Direct bil. |
| 12 | rs11563251 | T | C | -0.0242 | 0.0000 | 0.086 | 0.016 | 0.9981 | 0.001 | 1.03E-127 | Direct bil. |
| 13 | rs11569142 | T | G | 0.0045 | -0.0094 | 0.249 | 0.013 | 0.4714 | 0.001 | 5.77E-12 | Direct bil. |
| 14 | rs117011528 | A | G | -0.0148 | 0.0067 | 0.090 | 0.017 | 0.6948 | 0.001 | 3.23E-50 | Direct bil. |
| 15 | rs117846878 | G | T | -0.0067 | 0.0403 | 0.060 | 0.026 | 0.1259 | 0.001 | 2.52E-08 | Direct bil. |
| 16 | rs11890704 | C | T | 0.0046 | 0.0119 | 0.197 | 0.013 | 0.3680 | 0.001 | 1.07E-10 | Direct bil. |
| 17 | rs12228427 | G | A | 0.0172 | -0.0066 | 0.086 | 0.019 | 0.7275 | 0.001 | 6.65E-66 | Direct bil. |
| 18 | rs12308309 | G | C | -0.0044 | 0.0143 | 0.189 | 0.014 | 0.2999 | 0.001 | 1.14E-09 | Direct bil. |
| 19 | rs12766752 | C | T | -0.0033 | 0.0222 | 0.407 | 0.011 | 0.0382 | 0.001 | 1.63E-08 | Direct bil. |
| 20 | rs1292060 | A | G | 0.0033 | 0.0089 | 0.375 | 0.011 | 0.4097 | 0.001 | 2.36E-08 | Direct bil. |
| 21 | rs12988520 | C | A | 0.0289 | -0.0243 | 0.244 | 0.013 | 0.0573 | 0.001 | 1.00E-200 | Direct bil. |
| 22 | rs12996139 | A | C | 0.0090 | -0.0148 | 0.102 | 0.015 | 0.3199 | 0.001 | 7.61E-22 | Direct bil. |
| 23 | rs13018934 | G | A | 0.0034 | -0.0109 | 0.405 | 0.011 | 0.3238 | 0.001 | 3.32E-09 | Direct bil. |
| 24 | rs13030735 | A | C | -0.0136 | 0.0127 | 0.318 | 0.012 | 0.2737 | 0.001 | 6.17E-111 | Direct bil. |
| 25 | rs13289294 | T | C | 0.0037 | 0.0009 | 0.442 | 0.011 | 0.9343 | 0.001 | 1.18E-10 | Direct bil. |
| 26 | rs139116240 | C | T | -0.0305 | 0.0359 | 0.058 | 0.026 | 0.1693 | 0.001 | 1.73E-140 | Direct bil. |
| 27 | rs144708372 | T | C | 0.0100 | -0.0228 | 0.055 | 0.022 | 0.2955 | 0.001 | 1.05E-15 | Direct bil. |
| 28 | rs151075899 | T | G | 0.0077 | -0.0214 | 0.182 | 0.015 | 0.1487 | 0.001 | 2.23E-25 | Direct bil. |
| 29 | rs1597944 | C | T | 0.0353 | 0.0036 | 0.465 | 0.011 | 0.7488 | 0.001 | 1.00E-200 | Direct bil. |
| 30 | rs1654774 | A | G | 0.0038 | -0.0122 | 0.382 | 0.011 | 0.2686 | 0.001 | 6.44E-11 | Direct bil. |
| 31 | rs1661052 | G | A | 0.0127 | 0.0585 | 0.090 | 0.021 | 0.0057 | 0.001 | 2.12E-37 | Direct bil. |
| 32 | rs17866592 | C | T | -0.0065 | -0.0088 | 0.073 | 0.018 | 0.6343 | 0.001 | 2.58E-09 | Direct bil. |
| 33 | rs17868401 | A | G | 0.0039 | -0.0030 | 0.304 | 0.012 | 0.8000 | 0.001 | 1.39E-10 | Direct bil. |
| 34 | rs2068888 | G | A | -0.0042 | 0.0346 | 0.271 | 0.011 | 0.0016 | 0.001 | 5.20E-11 | Direct bil. |
| 35 | rs213554 | G | A | 0.0076 | -0.0134 | 0.484 | 0.011 | 0.2125 | 0.001 | 1.75E-40 | Direct bil. |
| 36 | rs2174011 | G | A | -0.0107 | 0.0236 | 0.317 | 0.012 | 0.0484 | 0.001 | 1.38E-69 | Direct bil. |
| 37 | rs2199766 | G | A | 0.0090 | -0.0058 | 0.434 | 0.011 | 0.5887 | 0.001 | 5.75E-56 | Direct bil. |
| 38 | rs2241883 | C | T | 0.0042 | 0.0067 | 0.217 | 0.013 | 0.5957 | 0.001 | 6.64E-10 | Direct bil. |
| 39 | rs2304776 | T | C | -0.0089 | -0.0016 | 0.338 | 0.012 | 0.8974 | 0.001 | 2.04E-50 | Direct bil. |
| 40 | rs2417977 | C | T | 0.0037 | -0.0098 | 0.260 | 0.012 | 0.4117 | 0.001 | 1.55E-08 | Direct bil. |
| 41 | rs28900682 | A | C | -0.0059 | -0.0208 | 0.096 | 0.021 | 0.3119 | 0.001 | 7.06E-10 | Direct bil. |
| 42 | rs28969670 | C | T | 0.0208 | -0.0096 | 0.070 | 0.024 | 0.6876 | 0.001 | 2.31E-78 | Direct bil. |
| 43 | rs2954021 | A | G | -0.0035 | 0.0024 | 0.429 | 0.011 | 0.8223 | 0.001 | 8.72E-10 | Direct bil. |
| 44 | rs36075906 | T | C | 0.0220 | -0.0094 | 0.253 | 0.013 | 0.4835 | 0.001 | 1.00E-200 | Direct bil. |
| 45 | rs3764043 | T | C | -0.0044 | 0.0282 | 0.200 | 0.017 | 0.0885 | 0.001 | 4.07E-10 | Direct bil. |
| 46 | rs4663580 | T | C | 0.0377 | -0.0516 | 0.054 | 0.027 | 0.0522 | 0.001 | 1.00E-200 | Direct bil. |
| 47 | rs502321 | A | T | -0.0092 | 0.0249 | 0.062 | 0.023 | 0.2730 | 0.001 | 4.86E-15 | Direct bil. |
| 48 | rs551118 | C | G | 0.0045 | -0.0112 | 0.353 | 0.011 | 0.3149 | 0.001 | 4.42E-14 | Direct bil. |
| 49 | rs55686299 | G | T | 0.0142 | -0.0042 | 0.066 | 0.024 | 0.8614 | 0.001 | 5.21E-36 | Direct bil. |
| 50 | rs55776147 | A | G | 0.0039 | 0.0137 | 0.233 | 0.014 | 0.3325 | 0.001 | 4.19E-09 | Direct bil. |
| 51 | rs56404001 | T | C | 0.0048 | -0.0036 | 0.184 | 0.015 | 0.8112 | 0.001 | 4.19E-11 | Direct bil. |
| 52 | rs567988934 | G | T | -0.0143 | -0.0062 | 0.056 | 0.026 | 0.8098 | 0.001 | 7.07E-31 | Direct bil. |
| 53 | rs634559 | A | G | 0.0057 | 0.0130 | 0.150 | 0.013 | 0.3220 | 0.001 | 5.45E-13 | Direct bil. |
| 54 | rs6749496 | C | T | 0.0663 | -0.0267 | 0.124 | 0.017 | 0.1149 | 0.001 | 1.00E-200 | Direct bil. |
| 55 | rs7260044 | T | C | 0.0041 | -0.0301 | 0.378 | 0.011 | 0.0082 | 0.001 | 3.21E-12 | Direct bil. |
| 56 | rs7310077 | G | A | 0.0070 | -0.0272 | 0.181 | 0.014 | 0.0560 | 0.001 | 1.10E-21 | Direct bil. |
| 57 | rs7591535 | T | A | 0.0149 | -0.0474 | 0.068 | 0.028 | 0.0919 | 0.001 | 1.01E-39 | Direct bil. |
| 58 | rs7595856 | G | A | 0.0150 | -0.0097 | 0.164 | 0.014 | 0.4757 | 0.001 | 4.68E-86 | Direct bil. |
| 59 | rs7603146 | A | G | -0.0038 | 0.0047 | 0.432 | 0.011 | 0.6575 | 0.001 | 3.94E-11 | Direct bil. |
| 60 | rs76159953 | C | T | -0.0083 | 0.0634 | 0.075 | 0.028 | 0.0230 | 0.001 | 1.62E-14 | Direct bil. |
| 61 | rs77768175 | G | A | -0.0055 | -0.1226 | 0.162 | 0.014 | 0.0000 | 0.001 | 4.30E-09 | Direct bil. |
| 62 | rs79522608 | A | G | -0.0090 | -0.0071 | 0.083 | 0.020 | 0.7263 | 0.001 | 1.97E-18 | Direct bil. |
| 63 | rs8176671 | C | A | 0.0042 | 0.0060 | 0.219 | 0.014 | 0.6684 | 0.001 | 6.98E-10 | Direct bil. |
| 64 | rs9247 | T | C | 0.0042 | 0.0069 | 0.344 | 0.012 | 0.5484 | 0.001 | 1.17E-12 | Direct bil. |
| 65 | rs9411364 | C | A | -0.0037 | -0.0228 | 0.247 | 0.012 | 0.0574 | 0.001 | 1.12E-08 | Direct bil. |
