# Supplementary material for: Causal association between serum bilirubin and ischemic stroke: multivariable Mendelian randomization
Source: Epidemiol Health. 2024 Aug 19;46:e2024070. doi: 10.4178/epih.e2024070 (PMC11826012; doi:10.4178/epih.e2024070)
Supplement: Supplementary Material 7. — List of 99 SNPs and association with total bilirubin and ischemic stroke [file epih-46-e2024070-Supplementary-7.docx]

Supplementary Material 7. List of 99 SNPs and association with total bilirubin and ischemic stroke

|  | SNP | A1 | A2 | beta.x | beta.y | eaf.x | se.y | pval.y | se.x | pval.x | exposure |
| --- | --- | --- | --- | --- | --- | --- | --- | --- | --- | --- | --- |
| 1 | rs10169532 | T | C | 0.0388 | 0.0076 | 0.458 | 0.011 | 0.4739 | 0.001 | 5.44E-196 | Total bil. |
| 2 | rs10175949 | G | A | 0.0189 | 0.0075 | 0.103 | 0.017 | 0.6637 | 0.002 | 1.03E-18 | Total bil. |
| 3 | rs10466790 | T | C | -0.0235 | 0.0241 | 0.051 | 0.022 | 0.2648 | 0.003 | 1.33E-15 | Total bil. |
| 4 | rs10495928 | G | A | -0.0093 | -0.0101 | 0.201 | 0.014 | 0.4804 | 0.002 | 9.79E-09 | Total bil. |
| 5 | rs10743399 | G | A | 0.0634 | -0.0037 | 0.213 | 0.013 | 0.7708 | 0.002 | 1.00E-200 | Total bil. |
| 6 | rs10743414 | C | T | -0.0089 | -0.0030 | 0.349 | 0.011 | 0.7937 | 0.001 | 6.11E-11 | Total bil. |
| 7 | rs10841651 | T | C | 0.0288 | -0.0317 | 0.162 | 0.015 | 0.0358 | 0.002 | 8.65E-60 | Total bil. |
| 8 | rs10849824 | C | T | 0.0097 | 0.0083 | 0.212 | 0.014 | 0.5582 | 0.002 | 1.16E-09 | Total bil. |
| 9 | rs10899116 | T | C | 0.0090 | 0.0172 | 0.222 | 0.012 | 0.1452 | 0.002 | 8.69E-09 | Total bil. |
| 10 | rs10995527 | A | G | -0.0158 | 0.0054 | 0.402 | 0.011 | 0.6168 | 0.001 | 5.66E-33 | Total bil. |
| 11 | rs11045508 | G | A | -0.0136 | -0.0131 | 0.083 | 0.021 | 0.5374 | 0.002 | 8.03E-09 | Total bil. |
| 12 | rs11045913 | A | G | -0.0155 | -0.0118 | 0.403 | 0.011 | 0.2720 | 0.001 | 1.91E-31 | Total bil. |
| 13 | rs114114722 | A | G | 0.0210 | -0.0172 | 0.074 | 0.022 | 0.4266 | 0.002 | 2.99E-17 | Total bil. |
| 14 | rs11563069 | G | A | 0.0100 | -0.0102 | 0.385 | 0.011 | 0.3555 | 0.001 | 5.04E-14 | Total bil. |
| 15 | rs11563102 | A | C | 0.0403 | -0.0116 | 0.060 | 0.023 | 0.6168 | 0.003 | 1.49E-49 | Total bil. |
| 16 | rs11563214 | G | T | -0.0205 | 0.0024 | 0.233 | 0.012 | 0.8409 | 0.002 | 1.44E-40 | Total bil. |
| 17 | rs11569142 | T | G | 0.0092 | -0.0094 | 0.249 | 0.013 | 0.4714 | 0.002 | 8.78E-10 | Total bil. |
| 18 | rs11575839 | A | G | -0.0150 | 0.0389 | 0.066 | 0.040 | 0.3326 | 0.003 | 1.00E-08 | Total bil. |
| 19 | rs11614319 | A | C | 0.0085 | -0.0087 | 0.263 | 0.013 | 0.4930 | 0.001 | 8.42E-09 | Total bil. |
| 20 | rs11671326 | T | C | 0.0104 | -0.0297 | 0.378 | 0.011 | 0.0092 | 0.001 | 9.44E-15 | Total bil. |
| 21 | rs11688580 | A | G | -0.0119 | 0.0124 | 0.360 | 0.012 | 0.2932 | 0.001 | 1.85E-18 | Total bil. |
| 22 | rs117379305 | T | C | 0.0195 | -0.0208 | 0.058 | 0.025 | 0.3973 | 0.003 | 2.18E-12 | Total bil. |
| 23 | rs118169288 | A | G | 0.0159 | 0.0436 | 0.059 | 0.018 | 0.0152 | 0.003 | 8.80E-09 | Total bil. |
| 24 | rs11890704 | C | T | 0.0137 | 0.0119 | 0.197 | 0.013 | 0.3680 | 0.002 | 5.50E-17 | Total bil. |
| 25 | rs12038217 | T | C | 0.0101 | 0.0132 | 0.236 | 0.013 | 0.3109 | 0.002 | 3.86E-11 | Total bil. |
| 26 | rs12228427 | G | A | 0.0450 | -0.0066 | 0.086 | 0.019 | 0.7275 | 0.002 | 2.02E-84 | Total bil. |
| 27 | rs12308309 | G | C | -0.0127 | 0.0143 | 0.189 | 0.014 | 0.2999 | 0.002 | 1.68E-14 | Total bil. |
| 28 | rs12996139 | A | C | 0.0297 | -0.0148 | 0.102 | 0.015 | 0.3199 | 0.002 | 1.21E-43 | Total bil. |
| 29 | rs12997325 | G | A | -0.0309 | -0.0068 | 0.372 | 0.012 | 0.5640 | 0.001 | 1.63E-116 | Total bil. |
| 30 | rs13142655 | T | C | -0.0078 | 0.0068 | 0.327 | 0.011 | 0.5399 | 0.001 | 1.52E-08 | Total bil. |
| 31 | rs13289294 | T | C | 0.0109 | 0.0009 | 0.442 | 0.011 | 0.9343 | 0.001 | 1.00E-16 | Total bil. |
| 32 | rs13388025 | A | T | 0.0624 | -0.0081 | 0.118 | 0.018 | 0.6584 | 0.002 | 1.00E-200 | Total bil. |
| 33 | rs138961643 | A | G | 0.1188 | -0.0275 | 0.061 | 0.025 | 0.2668 | 0.003 | 1.00E-200 | Total bil. |
| 34 | rs144708372 | T | C | 0.0280 | -0.0228 | 0.055 | 0.022 | 0.2955 | 0.003 | 1.59E-22 | Total bil. |
| 35 | rs151075899 | T | G | 0.0249 | -0.0214 | 0.182 | 0.015 | 0.1487 | 0.002 | 1.64E-49 | Total bil. |
| 36 | rs1654774 | A | G | 0.0098 | -0.0122 | 0.382 | 0.011 | 0.2686 | 0.001 | 2.64E-13 | Total bil. |
| 37 | rs1661052 | G | A | 0.0360 | 0.0585 | 0.090 | 0.021 | 0.0057 | 0.002 | 8.36E-56 | Total bil. |
| 38 | rs17419676 | G | A | 0.0095 | -0.0046 | 0.213 | 0.015 | 0.7587 | 0.002 | 2.07E-09 | Total bil. |
| 39 | rs17866592 | C | T | -0.0200 | -0.0088 | 0.073 | 0.018 | 0.6343 | 0.003 | 1.52E-15 | Total bil. |
| 40 | rs17868401 | A | G | 0.0128 | -0.0030 | 0.304 | 0.012 | 0.8000 | 0.001 | 1.40E-19 | Total bil. |
| 41 | rs17869073 | A | C | -0.0085 | 0.0248 | 0.322 | 0.011 | 0.0289 | 0.001 | 9.63E-10 | Total bil. |
| 42 | rs180363 | C | T | -0.0093 | -0.0120 | 0.181 | 0.016 | 0.4569 | 0.002 | 3.55E-08 | Total bil. |
| 43 | rs1823803 | C | T | 0.0911 | -0.0019 | 0.101 | 0.025 | 0.9373 | 0.002 | 1.00E-200 | Total bil. |
| 44 | rs1840144 | A | T | 0.0902 | -0.0018 | 0.371 | 0.011 | 0.8731 | 0.001 | 1.00E-200 | Total bil. |
| 45 | rs185992704 | A | G | 0.0149 | 0.0033 | 0.079 | 0.018 | 0.8517 | 0.002 | 7.19E-10 | Total bil. |
| 46 | rs2068888 | G | A | -0.0117 | 0.0346 | 0.271 | 0.011 | 0.0016 | 0.001 | 1.05E-15 | Total bil. |
| 47 | rs213554 | G | A | 0.0229 | -0.0134 | 0.484 | 0.011 | 0.2125 | 0.001 | 7.40E-69 | Total bil. |
| 48 | rs2174011 | G | A | -0.0288 | 0.0236 | 0.317 | 0.012 | 0.0484 | 0.001 | 5.94E-95 | Total bil. |
| 49 | rs2199766 | G | A | 0.0242 | -0.0058 | 0.434 | 0.011 | 0.5887 | 0.001 | 4.04E-76 | Total bil. |
| 50 | rs2236683 | A | C | 0.0087 | -0.0177 | 0.245 | 0.013 | 0.1603 | 0.002 | 8.38E-09 | Total bil. |
| 51 | rs2302154 | C | T | 0.0165 | -0.0114 | 0.070 | 0.022 | 0.5973 | 0.003 | 1.10E-10 | Total bil. |
| 52 | rs2417861 | C | T | -0.0095 | -0.0153 | 0.224 | 0.013 | 0.2258 | 0.002 | 9.57E-10 | Total bil. |
| 53 | rs28766953 | A | G | -0.0133 | -0.0254 | 0.087 | 0.021 | 0.2201 | 0.002 | 9.57E-09 | Total bil. |
| 54 | rs2900459 | G | A | -0.0094 | -0.0143 | 0.430 | 0.012 | 0.2186 | 0.001 | 9.34E-13 | Total bil. |
| 55 | rs2971856 | A | G | 0.0300 | -0.0369 | 0.058 | 0.018 | 0.0419 | 0.003 | 3.87E-27 | Total bil. |
| 56 | rs4410790 | C | T | -0.0097 | 0.0093 | 0.401 | 0.011 | 0.3962 | 0.001 | 2.54E-13 | Total bil. |
| 57 | rs454139 | C | T | -0.0141 | 0.0122 | 0.114 | 0.020 | 0.5391 | 0.002 | 5.29E-12 | Total bil. |
| 58 | rs4598207 | T | A | 0.0078 | -0.0180 | 0.337 | 0.011 | 0.1027 | 0.001 | 1.25E-08 | Total bil. |
| 59 | rs4663245 | G | A | -0.0486 | 0.0152 | 0.421 | 0.011 | 0.1833 | 0.001 | 1.00E-200 | Total bil. |
| 60 | rs4737010 | G | A | 0.0089 | 0.0121 | 0.463 | 0.011 | 0.2885 | 0.001 | 9.93E-12 | Total bil. |
| 61 | rs4972193 | T | C | 0.0078 | 0.0028 | 0.395 | 0.011 | 0.7948 | 0.001 | 4.17E-09 | Total bil. |
| 62 | rs502321 | A | T | -0.0304 | 0.0249 | 0.062 | 0.023 | 0.2730 | 0.003 | 1.27E-29 | Total bil. |
| 63 | rs551118 | C | G | 0.0124 | -0.0112 | 0.353 | 0.011 | 0.3149 | 0.001 | 7.88E-20 | Total bil. |
| 64 | rs55671977 | G | C | -0.0119 | -0.0095 | 0.179 | 0.015 | 0.5328 | 0.002 | 2.03E-12 | Total bil. |
| 65 | rs55686299 | G | T | 0.0473 | -0.0042 | 0.066 | 0.024 | 0.8614 | 0.003 | 2.68E-73 | Total bil. |
| 66 | rs56404001 | T | C | 0.0104 | -0.0036 | 0.184 | 0.015 | 0.8112 | 0.002 | 5.19E-10 | Total bil. |
| 67 | rs567988934 | G | T | -0.0390 | -0.0062 | 0.056 | 0.026 | 0.8098 | 0.003 | 2.07E-43 | Total bil. |
| 68 | rs62192338 | A | G | -0.0121 | 0.0275 | 0.143 | 0.016 | 0.0789 | 0.002 | 8.21E-11 | Total bil. |
| 69 | rs62195072 | C | T | 0.0432 | -0.0052 | 0.322 | 0.012 | 0.6667 | 0.001 | 1.00E-200 | Total bil. |
| 70 | rs6431630 | A | G | -0.0733 | 0.0005 | 0.087 | 0.016 | 0.9749 | 0.002 | 1.00E-200 | Total bil. |
| 71 | rs6602905 | C | T | -0.0081 | 0.0025 | 0.295 | 0.013 | 0.8464 | 0.001 | 1.60E-08 | Total bil. |
| 72 | rs662799 | G | A | 0.0128 | -0.0181 | 0.296 | 0.011 | 0.1084 | 0.001 | 2.97E-19 | Total bil. |
| 73 | rs6717651 | G | A | -0.0094 | 0.0208 | 0.280 | 0.012 | 0.0834 | 0.001 | 7.81E-11 | Total bil. |
| 74 | rs6749496 | C | T | 0.2139 | -0.0267 | 0.124 | 0.017 | 0.1149 | 0.002 | 1.00E-200 | Total bil. |
| 75 | rs6779146 | C | T | 0.0200 | 0.0067 | 0.125 | 0.014 | 0.6293 | 0.002 | 3.90E-24 | Total bil. |
| 76 | rs7088405 | T | G | -0.0077 | -0.0023 | 0.369 | 0.011 | 0.8382 | 0.001 | 1.05E-08 | Total bil. |
| 77 | rs7310077 | G | A | 0.0181 | -0.0272 | 0.181 | 0.014 | 0.0560 | 0.002 | 6.69E-27 | Total bil. |
| 78 | rs73121253 | T | A | 0.0100 | -0.0223 | 0.155 | 0.016 | 0.1690 | 0.002 | 2.94E-08 | Total bil. |
| 79 | rs7419666 | C | T | 0.0089 | 0.0163 | 0.208 | 0.015 | 0.2664 | 0.002 | 2.35E-08 | Total bil. |
| 80 | rs74368849 | A | G | 0.0151 | 0.0008 | 0.078 | 0.020 | 0.9702 | 0.002 | 5.21E-10 | Total bil. |
| 81 | rs74557947 | A | T | -0.0246 | -0.0134 | 0.071 | 0.020 | 0.5063 | 0.003 | 2.58E-22 | Total bil. |
| 82 | rs75544767 | C | T | 0.0211 | 0.0132 | 0.062 | 0.026 | 0.6141 | 0.003 | 3.44E-15 | Total bil. |
| 83 | rs7569837 | C | T | -0.0084 | -0.0098 | 0.441 | 0.011 | 0.3565 | 0.001 | 1.24E-10 | Total bil. |
| 84 | rs75721934 | G | A | 0.0082 | 0.0010 | 0.285 | 0.013 | 0.9379 | 0.001 | 1.46E-08 | Total bil. |
| 85 | rs7603146 | A | G | -0.0128 | 0.0047 | 0.432 | 0.011 | 0.6575 | 0.001 | 1.46E-22 | Total bil. |
| 86 | rs76159953 | C | T | -0.0155 | 0.0634 | 0.075 | 0.028 | 0.0230 | 0.002 | 3.04E-10 | Total bil. |
| 87 | rs76518000 | G | A | -0.0969 | 0.0331 | 0.058 | 0.026 | 0.2082 | 0.003 | 1.00E-200 | Total bil. |
| 88 | rs7741443 | T | A | -0.0116 | 0.0405 | 0.119 | 0.018 | 0.0218 | 0.002 | 8.61E-09 | Total bil. |
| 89 | rs77768175 | G | A | -0.0176 | -0.1226 | 0.162 | 0.014 | 0.0000 | 0.002 | 2.64E-23 | Total bil. |
| 90 | rs7828742 | A | G | 0.0079 | -0.0047 | 0.409 | 0.011 | 0.6663 | 0.001 | 2.20E-09 | Total bil. |
| 91 | rs78614326 | A | G | -0.0197 | 0.0393 | 0.053 | 0.028 | 0.1576 | 0.003 | 8.49E-12 | Total bil. |
| 92 | rs79260437 | T | C | -0.0102 | 0.0119 | 0.146 | 0.016 | 0.4580 | 0.002 | 3.07E-08 | Total bil. |
| 93 | rs7947951 | G | A | -0.0094 | -0.0115 | 0.313 | 0.012 | 0.3282 | 0.001 | 1.76E-11 | Total bil. |
| 94 | rs877908 | C | T | 0.0089 | -0.0110 | 0.497 | 0.011 | 0.3027 | 0.001 | 7.92E-12 | Total bil. |
| 95 | rs906747 | G | A | 0.0099 | -0.0203 | 0.168 | 0.014 | 0.1499 | 0.002 | 1.45E-08 | Total bil. |
| 96 | rs9247 | T | C | 0.0107 | 0.0069 | 0.344 | 0.012 | 0.5484 | 0.001 | 3.66E-15 | Total bil. |
| 97 | rs9272226 | C | T | 0.0074 | -0.0215 | 0.442 | 0.011 | 0.0492 | 0.001 | 1.55E-08 | Total bil. |
| 98 | rs9379895 | G | A | -0.0105 | 0.0379 | 0.155 | 0.014 | 0.0086 | 0.002 | 6.42E-09 | Total bil. |
| 99 | rs961169 | T | C | 0.0080 | 0.0141 | 0.437 | 0.011 | 0.1861 | 0.001 | 9.50E-10 | Total bil. |
