# Supplementary material for: Causal association between serum bilirubin and ischemic stroke: multivariable Mendelian randomization
Source: Epidemiol Health. 2024 Aug 19;46:e2024070. doi: 10.4178/epih.e2024070 (PMC11826012; doi:10.4178/epih.e2024070)
Supplement: Supplementary Material 8. — Sensitivity analysis in MR regarding bilirubin and ischemic stroke [file epih-46-e2024070-Supplementary-8.docx]

Supplementary Material 8. Sensitivity analysis in MR regarding bilirubin and ischemic stroke

|  | nsnp | b | se | p-value | lo_ci | up_ci | or | or_lci95 | or_uci95 |
| --- | --- | --- | --- | --- | --- | --- | --- | --- | --- |
| Total bilirubin |  |  |  |  |  |  |  |  |  |
| MR Egger | 99 | -0.08131 | 0.084922 | 0.340719 | -0.24775 | 0.085138 | 0.921909 | 0.780551 | 1.088867 |
| Weighted median | 99 | -0.12317 | 0.07009 | 0.078865 | -0.26055 | 0.014206 | 0.884114 | 0.770631 | 1.014308 |
| Weighted mode | 99 | -0.11914 | 0.056917 | 0.038909 | -0.2307 | -0.00758 | 0.887682 | 0.793979 | 0.992444 |
|  |  |  |  |  |  |  |  |  |  |
| Direct bilirubin |  |  |  |  |  |  |  |  |  |
| MR Egger | 65 | -0.3493 | 0.285184 | 0.225197 | -0.90826 | 0.209657 | 0.705179 | 0.403224 | 1.233255 |
| Weighted median | 65 | -0.40237 | 0.205849 | 0.050619 | -0.80583 | 0.001093 | 0.668733 | 0.446715 | 1.001093 |
| Weighted mode | 65 | -0.39158 | 0.160907 | 0.017753 | -0.70696 | -0.0762 | 0.675988 | 0.493142 | 0.926628 |
|  |  |  |  |  |  |  |  |  |  |
| Indirect bilirubin |  |  |  |  |  |  |  |  |  |
| MR Egger | 79 | -0.09268 | 0.125435 | 0.462214 | -0.33854 | 0.153169 | 0.911481 | 0.712812 | 1.165522 |
| Weighted median | 79 | -0.10655 | 0.09084 | 0.240833 | -0.28459 | 0.071499 | 0.898933 | 0.752321 | 1.074117 |
| Weighted mode | 79 | -0.13504 | 0.072822 | 0.067461 | -0.27777 | 0.00769 | 0.87368 | 0.75747 | 1.007719 |
