# Supplementary material for: Causal association between serum bilirubin and ischemic stroke: multivariable Mendelian randomization
Source: Epidemiol Health. 2024 Aug 19;46:e2024070. doi: 10.4178/epih.e2024070 (PMC11826012; doi:10.4178/epih.e2024070)
Supplement: Supplementary Material 9. — LocusZoom plots illustrating no evidence of genetic colocalization between indirect bilirubin at the SLCO1B3 gene locus and ischemic stroke risk at the SLCO1B3 gene locus. [file epih-46-e2024070-Supplementary-9.docx]

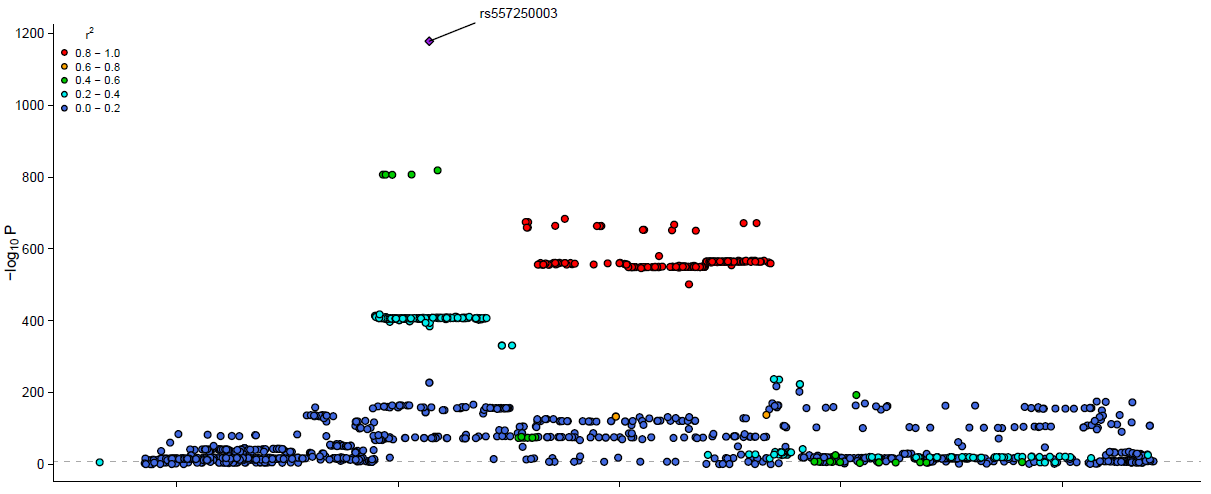


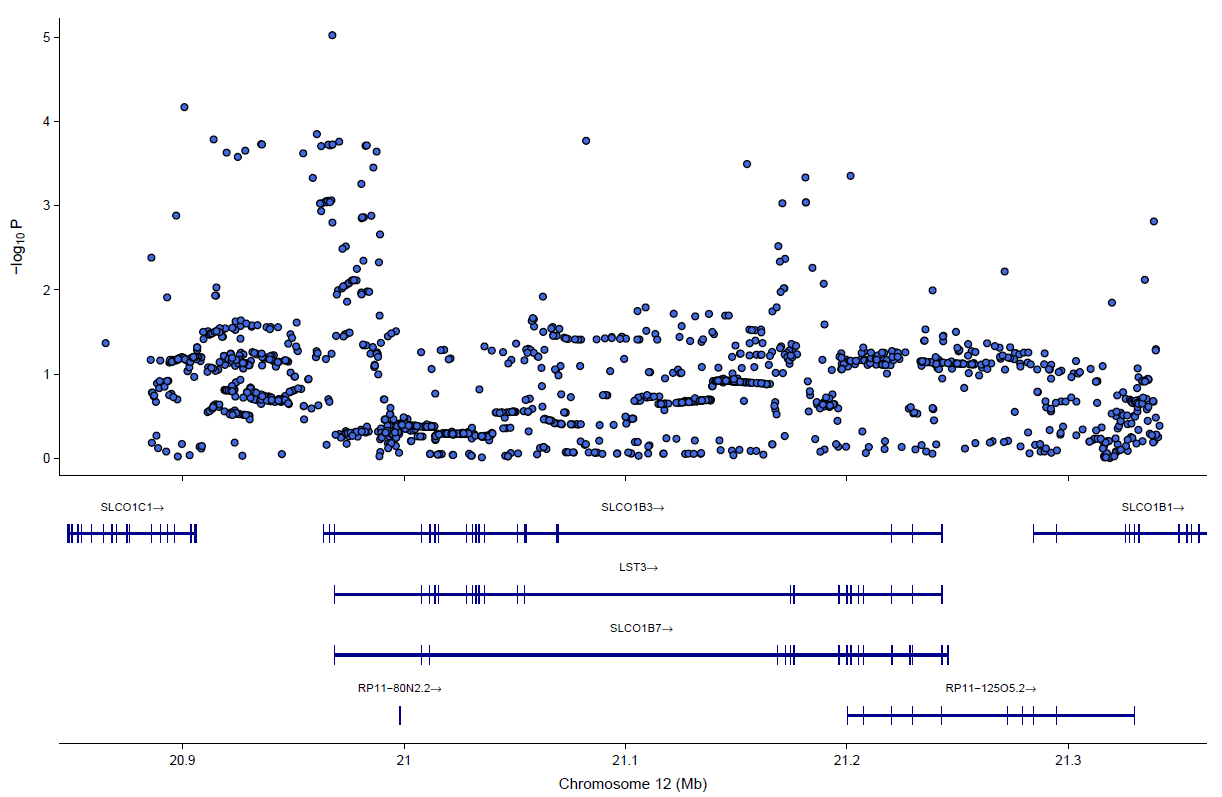


Supplementary Material 9. LocusZoom plots illustrating no evidence of genetic colocalization between indirect bilirubin at the *SLCO1B3* gene locus and ischemic stroke risk at the *SLCO1B3* gene locus.
